# Large-scale resurgence of herpangina in Japan, following the easing of COVID-19 measures

**DOI:** 10.1016/j.ijregi.2023.11.008

**Published:** 2023-11-14

**Authors:** Mugen Ujiie, Takato Nakamoto, Noriko Iwamoto, Shinya Tsuzuki

**Affiliations:** National Center for Global Health and Medicine, Tokyo, Japan

**Keywords:** Herpangina, COVID-19 pandemic, Resurgence, Japan

## Abstract

**Objectives:**

This study examined the rise in herpangina cases and its demographic trends in Japan post-easing of COVID-19 restrictions in the summer of 2023, assessing its potential strain on pediatric healthcare.

**Methods:**

Weekly herpangina cases data from the National Institute of Infectious Diseases from 2017 to 2023 were analyzed using difference-in-differences methodology and Poisson regression models, incorporating variables such as time, the COVID-19 pandemic, and the easing of measures. Statistical significance was set at a two-sided *P*-value of 0.05.

**Results:**

A significant increase in herpangina cases was observed from the 19th to the 27th week of 2023, with a notable shift in age distribution compared to that of pre-pandemic years. Poisson regression analysis revealed a positive correlation between the number of herpangina cases and the easing of infection control measures. There was an increase in other respiratory illnesses, including respiratory syncytial virus, particularly in children aged 3 and above.

**Conclusion:**

We found a surge in herpangina cases in Japan after the easing of the COVID-19 restrictions during the summer of 2023, along with a noticeable increase in other respiratory illnesses, emphasizing the potential strain on pediatric healthcare and reinforcing the ongoing significance of implementing general infection prevention strategies.

## Highlights

• Herpangina surges in Japan post-COVID-19 measure relaxation.
• The increase is most notable in children aged 3 years and older.
• Number of herpangina cases is related with the easing of infection control measures.
• Age distribution differs significantly from pre-pandemic years.
• The findings underscore the need for ongoing universal infection prevention.

On May 05, 2023, the World Health Organization officially stated that COVID-19 no longer constitutes a public health emergency of international concern. Moreover, the Japanese government reclassified the disease from “Novel Influenza and Others” to a Class 5 Infectious Disease on May 08, 2023, akin to seasonal influenza. These changes have led to a significant shift in Japan's public health response, including easing isolation measures for COVID-19 and transitioning from universal to sentinel surveillance [1]. Consequently, while public funding for COVID-19 testing has decreased, the level of SARS-CoV-2 testing has remained consistent [2]. However, based on reports from approximately 5000 sentinel medical institutions nationwide, the average number of SARS-CoV-2 positive cases per sentinel site has continuously risen from 2.63 in week 19 (May 08-14, 2023) to 20.50 in week 35 (August 28-September 03, 2023) [2], suggesting potential epidemic expansion following the relaxation of infection control measures. Additionally, reports of non-SARS-CoV-2 viral infections such as respiratory syncytial virus (RSV) and influenza have increased, raising concerns regarding the broad impact of COVID-19 mitigation strategies [3,4]. Here, we report an increased incidence of herpangina in Japan following these changes.

Herpangina is an acute viral pharyngitis primarily caused by the enterovirus group, predominantly the Coxsackie A virus. Similarly, Coxsackie B and echoviruses can trigger the disease. Transmission of the virus occurs through fecal-oral routes including contact transmission, and respiratory droplets. Notably, herpangina predominantly affects infants and young children, peaking from early summer to fall. In Japan, herpangina is classified as a notifiable disease in pediatric institutions based on the Infectious Diseases Control Law. Importantly, the diagnostic criteria encompass clinical features, the abrupt onset of high fever, and the presence of vesicles, ulcers, or redness in the vicinity of the uvula.

We utilized data from the National Institute of Infectious Diseases (NIID) from 2017 to 2022 to compare weekly herpangina activity in the 2023 season with that in the previous six seasons [5]. Specifically, the NIID collects weekly reports of herpangina cases diagnosed by pediatricians from approximately 3000 sentinel centers, including hospitals and clinics, across all 47 prefectures. Our analysis covers data up to week 34 of 2023. We used Pearson's chi-square test to examine the patients’ age distribution difference between this year (2023) and pre-pandemic era (the average of 2017, 2018, and 2019). Moreover, a comparison was performed using the difference-in-differences methodology [6], assuming potential influences from the strengthening of COVID-19 measures in 2020 and the easing of measures from the 19th week of 2023. Notably, variables such as time (weeks), the COVID-19 pandemic, and the easing of measures were integrated into a Poisson regression model, taking into account interactions between COVID-19 and year, COVID-19 and weeks, and the easing of measures and weeks. Analyses were conducted using R (version 4.3.0), with statistical significance set at a two-sided *P*-value of 0.05.

**Figure 1:**
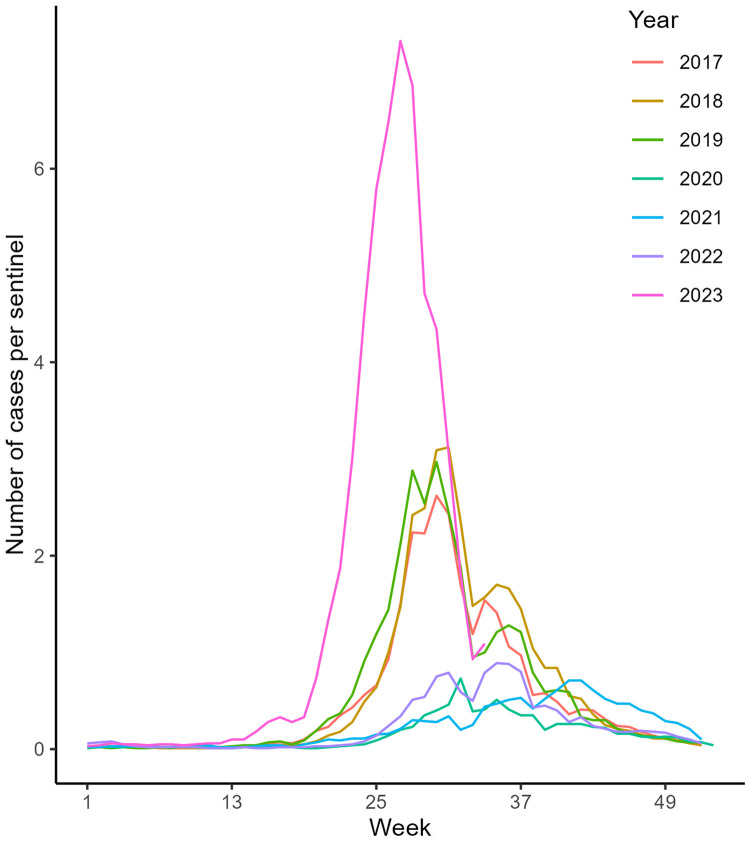
Herpangina in children, by year and epidemiological week, Japan, January 2017–Aug 2023 (as of epidemiological week 34, 2023).

The number of herpangina cases per sentinel medical institution notably increased from 0.33 in the 19th week of 2023 to 7.32 in the 27th week, compared with that in previous years. (Figure 1). Furthermore, Poisson regression analysis revealed a positive correlation between the herpangina reports number and the number of weeks, indicating the typical herpangina seasonal pattern in summer (Table 1). Finally, a positive correlation was observed between the herpangina reports number and the easing of infection control measures, suggesting an impact of the relaxation of measures.

**Table 1:** Results of Poisson regression analysis.

Variable	Incidence rate ratio	95% confidence interval	*P*-value
Week	1.18	1.18-1.18	<0.001
Year	1.07	1.07-1.08	<0.001
COVID-19	0.0	0.0-0.0	<0.001
Easing of infection control measures	127.29	121.93-132.90	<0.001
Interaction term (COVID-19 * Week)	0.96	0.96-0.96	<0.001
Interaction term (COVID-19 * Year)	1.35	1.33-1.37	<0.001
Interaction term (Easing of infection control measures * Week)	0.90	0.90-0.90	<0.001

Based on the NIID's evaluation up to week 28, the age-group proportions in cumulative reports of herpangina patients for 2023 indicated that the percentages for ages 3, 4, and 5 were 16.7%, 14.8%, and 10.8%, respectively [7], representing an increase compared to the pre-COVID-19 pandemic 3-year average from 2017–2019 of 13.3%, 9.5%, and 5.7%, respectively. Furthermore, Pearson's chi-square test revealed that the proportion of cases among ages 3, 4, and 5 in 2023 was significantly higher than that in pre-pandemic years (*P* <0.001); conversely, those for ages 0 and 1 were 6.0% and 20.7%, respectively, decreasing from the pre-pandemic 3-year average of 10.3% and 31.5%, respectively (*P* <0.001). Moreover, in 2023, the percentage of patients aged 5 and below was 88.2%, lower than the pre-pandemic 3-year average of 91.8% (*P* <0.001). Thus, considering the decline in reported cases from 2020 to 2022, the changes in age proportions in 2023 suggest the effects of enhanced infection control measures during the COVID-19 pandemic, such as wearing masks indoors and adhering to the '3Cs' guidelines (avoiding crowded places, close-contact settings, and confined and enclosed spaces) [8]. Additionally, the accumulation of susceptible individuals as a result of the decreased infection incidence may also contribute to the observed epidemiological trends, alongside the subsequent relaxation of these measures in 2023. Similarly, in reports of RSV infections, we observed a trend in which the age distribution of individuals infected during the resurgence shifted to older age groups compared to the age distribution before the pandemic [3].

The type and distribution of viruses detected from herpangina patients vary annually. According to the NIID's Pathogen Detection Information System, the predominant viruses isolated and detected from herpangina patients in the past 5 years were, in descending order: Coxsackievirus A6 (CA6) followed by CA5 in 2019, CA4 followed by CA2 in 2020, CA4 followed by CA6 in 2021, and CA6 followed by CA4 and CA2 in 2022. As of September 8, 2023, out of 368 cases, nearly half were attributed to CA2.

To explore the impact of easing infection control measures, we also examined trends in other representative viral respiratory infections. In Japan, the influenza activity over the past decade typically saw the number of patients per sentinel medical institution drop to less than 0.2 during the summer. However, this year, continuous reports of more than 1.0 patients were observed from the 19th week, registering 1.36 patients, through to the 34th week, indicating the potential influence of system changes in public health policies, healthcare practices, and community behaviors in response to the COVID-19 pandemic. Similarly, for RSV, the number of patients per sentinel increased from 1.04 in the 19th week to 7.32 in the 27th week, indicating a significant expansion of the outbreak, akin to the trend observed with herpangina.

For infants aged ≤ 5 years hospitalized with SARS-CoV-2 infection, the disease severity increases with coinfection with respiratory viruses, including RSV and rhinovirus/enterovirus [9,10]. Therefore, in addition to the rare complications of herpangina, such as aseptic meningitis and acute myocarditis, care must be taken to avoid overlooking coinfections.

A limitation of this report is that the post-19th week rise in cases is inconsistent across all diseases mandated for reporting under the Infectious Diseases Control Law, necessitating clarification for the pronounced increase in herpangina cases. Moreover the reporting system is confined to pediatric medical institutions; leaving adult infection cases unrecorded.

Since Japan relaxed COVID-19 measures, herpangina incidence has risen, particularly in children aged 3 and older, whose infections were suppressed during the pandemic. Vigilance is needed to prevent coinfections with COVID-19 in infants, as concurrent pediatric infectious diseases could strain healthcare, impeding proper care. Hence, even after easing COVID-19 measures, essential infection control practices, such as hand hygiene and mask-wearing, should continue.

## Declaration of competing interest

The authors have no competing interests to declare.
